# Flawless victory! Investigating search and experience qualities as antecedent predictors of video game success

**DOI:** 10.1007/s12525-023-00647-2

**Published:** 2023-05-19

**Authors:** Sven Heidenreich, Franziska Handrich, Tobias Kraemer

**Affiliations:** 1 Faculty of Human and Business Sciences, Saarland University, Building C3 1, 66123 Saarbruecken, Saarland, Germany; 2 Institute for Management, University of Koblenz, Universitätsstraße 1, 56070 Koblenz, Germany

**Keywords:** Video games, Success factors, Search qualities, Experience qualities, L86, O30

## Abstract

In recent years, video games have been on the rise as entertainment goods, leading to a growing interest by practitioners, researchers, and, of course, consumers alike. While a few unusually successful video games produce overall high revenues, most released games struggle to break even. Hence, there is an urgent need to better understand what distinguishes financially successful games from nonsuccessful video games. Accordingly, several researchers have called for investigations into the drivers of the financial success of video games. However, empirical studies within this respect are still lacking. Based on longitudinal data of 351 video games, the current study strives to fill this research gap by investigating the relative importance of potential success factors for the short-term and long-term financial success of video games. The results of multiple regression analyses confirm that search qualities such as brand popularity, reviews, and awards as well as experience qualities such as graphics, sound, and game duration significantly drive financial success in terms of the total number of sold video games in Europe. Consequently, managers in the video game industry can boost their chances for the production of a successful video game by focusing on these factors.

## Introduction

The video game industry generated US$174 billion in worldwide revenue in 2020, which represents a growth of 19.6% from the previous year (Ullmann et al., 2022). For young people between the ages of 14 and 29, playing video games is currently one of the most popular entertainment outlets available (Marchand, 2016). As a consequence, the video game industry has already achieved sales equal to or exceeding those of other large entertainment sectors, such as the movie industry (Choi et al., 2018; Hamari & Keronen, 2017; Subramanian et al., 2011). In general, the production of a video game requires high investments ranging from, e.g., 56.7 € million (Heavy Rain, 2010) to 316 € million (Cyberpunk 2077, 2020), including development and marketing budgets. However, only a few games, often so-called blockbuster games (i.e., unusually successful video games with widespread popularity and a very large number of sales), make up the majority of sold items per year, return the investments made, and produce overall high revenues. However, most released games struggle to break even (Cox, 2014; Ullmann et al., 2022). Subsequently, this situation leads to a high risk for producers and developers of games alike. Accordingly, the need to better understand what distinguishes successful games from nonsuccessful games, in terms of copies sold, is increasing, making the video game industry and its success factors a highly relevant research topic.

Despite the relevance of video games for the entertainment industry (Koivisto & Hamari, 2019), only a few studies have been conducted on this research topic (e.g., Borowiecki & Prieto-Rodriguez, 2015; Choi et al., 2018; Handrich et al., 2022; Harviainen et al., 2018; Koch & Bierbamer, 2016; Marchand, 2016), with most of the research concentrating on the possible effects game playing can have on game players (Siemens et al., 2015; Wang et al., 2021; Zhang et al., 2019). While there have been several calls for research on drivers of video game success (Handrich et al., 2022; Marchand & Hennig-Thurau, 2013; Poelset al., 2012), empirical studies within this respect are scarce (Marchand, 2016; Pfau et al., 2022; Ullmann et al., 2022). Thus, research in this area is needed. In contrast to the video game industry, research concerning success factors in the entertainment industry is not a new topic. For example, several studies have identified success factors for the motion picture industry. Among the most important factors were content (Austin, 1981), awards (Hennig-Thurau et al., 2001), star factor (Hennig-Thurau et al., 2012), and production costs (Gemser et al., 2007). As video games have a close link with motion pictures, both are so-called hedonic products and part of the entertainment industry (Kim & Han, 2009), it seems reasonable to assume that at least some of these factors might also play an important role in generating financial success for video games. However, unlike other forms of entertainment products, video games incorporate both audio and visual components and simultaneously require users to engage with content in complex ways (Tavinor, 2008). Hence, it remains subject to debate whether and how previously identified success factors of entertainment products might also be relevant for the financial success of video games. Furthermore, it is also unknown whether success factors for video games exist that are not already covered by previously identified success factors of other entertainment products such as movies. Since video games differ essentially from other entertainment products, there is a lack of additional success factors that are defined for the peculiarities of the video game industry. Finally, it also lacks a theoretical framework for potential success factors in the entertainment industry generally and for video games specifically because of the following shortcomings in prior studies. Prior research, mostly conducted in the movies industry, has focused on the sole investigation of one or a constrained set of success factors (e.g., Hennig-Thurau et al., 2001, 2012). As a result, studies with a comprehensive set of success factors are lacking, enabling the simultaneous determination of the relative importance of each factor in achieving financial success. Perhaps as a result, previously identified success drivers are derived more or less loosely side by side without any theoretical anchoring in terms of an established framework that might also help classify such success factors.

The current study is among the first to address these research gaps by investigating the relative importance of potential success factors of video games and thereby advances the research in three ways. First, previously identified success factors in neighboring sectors of the entertainment industry are screened for transferability to the video games sector and subsequently empirically validated for effectiveness. Based on this validation, this study provides the first empirical evidence on the transferability of success drivers from other entertainment sectors to the context of video games, enhancing the external validity of established success factors. Second, based on theoretical considerations, additional success factors that are tailor-made for the peculiarities of the video game industry and were not already covered by previous research in neighboring areas are derived. By identifying and empirically validating video game-specific success factors, this study extends previous knowledge on the success drivers of entertainment products and provides a more comprehensive picture in this regard. Third, based on the theory of economics of information (Hirshleifer & Riley, 1992; Nelson, 1970; Stigler, 1961), established and newly identified success drivers are theoretically anchored in a newly formulated framework based on their search and experience qualities. In doing so, this study offers a theoretical framework for video game success drivers, serving as a basis for future research to identify additional success factors in the entertainment industry and expand upon this framework.

The remainder of this manuscript is organized as follows: The next section provides a brief overview of previously identified success factors in the entertainment industry, along with theoretical considerations of potential but previously unexplored success factors that could be relevant in the context of video games. Based on insights from this theoretical consolidation, a typology of potential success factors for video games is developed, which differentiates search from experience qualities as main drivers for video game success based on the theory of economics of information (Hirshleifer & Riley, 1992; Nelson, 1970; Stigler, 1961). Subsequently, hypotheses on the effects of search and experience qualities on the short- and long-term financial success of video games are derived and empirically analyzed using longitudinal data of 351 computer games. More specifically, primary and secondary data for computer games published between 2010 and 2015 were collected from different sources (internet databases, magazines, expert opinions) to operationalize our independent variables. For our dependent variable, we followed prior research in the video game sector (Cox, 2014; Handrich et al., 2022; Situmeang et al., 2016) and measured video game success through sales figures in terms of all units sold over a specific period of time. Based on this dataset, multiple regression analyses were run to test our research models. This article concludes with a discussion of both theoretical and practical implications, as well as limitations and future research avenues.

## A typology of video game success factors

In line with previous research in the video game industry (Cox, 2014; Handrich et al., 2022; Situmeang et al., 2016), video game success in this study is understood as the number of all sold units during a specific duration of time. As previously mentioned in the “Introduction” section, research concerning success factors within the video game industry has, to the best of the authors’ knowledge, been scarce. However, for other entertainment products, especially motion pictures, information about possible success factors is readily available and has been empirically researched numerous times before. As all entertainment products are experience goods for which the complete value can only be assessed after buying the product (Choi & Kim, 2004; Deuchert et al., 2005; Hennig-Thurau et al., 2001; Reinstein & Snyder, 2005), existing success factors of entertainment products might be to a certain extent adaptable to the video game industry. However, not all success factors identified in other entertainment products are transferable to video games, as they uniquely combine audio-visual elements with complex user engagement (Tavinor, 2008). Additionally, and even more importantly, additional success factors that are specific to the peculiarities of the video game industry might have not already been covered by previous research in neighboring areas. Since plenty of success factors in the entertainment industry have emerged over time and might even be extended by video game-specific success factors, a theoretical framework is needed for structuring and theoretical anchoring. Within this respect, a classification into search and experience qualities as certain types of success factors based on economics of information theory seems beneficial (Hirshleifer & Riley, 1992; Nelson, 1970; Stigler, 1961).

### Theory of economics of information

The theory of economics of information deals with the study of information in economic decision-making and has its roots in political economy (Stigler, 1961; Nelson, 1970). The main focus of the economics of information theory relies on how information is acquired, processed, and disseminated, and how corresponding information search processes influence the behavior of individuals and companies in the market (Nelson, 1970). One central aspect of this theory is the distinction “between two fundamental processes of acquiring information: search or experience” (Sichtmann, 2007 p. 62). Accordingly, economics of information theory distinguishes between two types of product characteristics based on how the information about these characteristics is acquired: search and experience qualities (Hennig-Thurau et al., 2001; Nelson, 1970). Experience qualities refer to the characteristics of products that can only be described on consumption, whereas search qualities of products refer to information that is available immediately after product release (Hirshleifer & Riley, 1992). The distinction between search and experience qualities is important because the way consumers gather information and make purchasing decisions differs depending on the type of quality attribute. For search qualities, consumers can rely on easily accessible information, such as product descriptions, reviews, and ratings. For experience quality attributes, however, consumers must rely on their own experience or the experience of others who have used the product (Nelson, 1970; Sichtmann, 2007). In this respect, the theory of economics of information suggests that the availability and quality of information can have a significant impact on consumer behavior (Hennig-Thurau et al., 2001). If consumers have limited information about the true quality of a product, they might be hesitant to make a purchase, or they may even refrain from a purchase because of incomplete or inaccurate information. Especially for complex products or services, such as video games, success heavily depends on the ease with which customers can find the information they need about a product or service (Maute and Forrester, 1991; Hennig-Thurau et al., 2001). Since search and experience qualities significantly vary in terms of ease with which customers can find related information, the contribution of certain video game attributes to success might thus result from a dominance of either search or experience qualities in these attributes. Hence, using the dichotomy of search and experience qualities from economies of information theory to derive a typology of video game success factors might help to better understand why some video games are successful and others are not. Accordingly, potential success factors of video games are discussed in the following, starting with an assignment to either search or experience qualities based on how the information about these factors is acquired and concluding with a respective hypothesis on how the potential success factor affects video game success.

### Search qualities and video game success

Following economies of information theory, information about the search qualities of video games (e.g., ratings, production costs, player modes) is readily available to consumers prior to purchase without consuming the respective offer (Hennig-Thurau et al., 2001). Hence, acquiring information about search qualities proceeds without large effort on the part of the consumer (both cognitive and financial; Maute and Forrester, 1991). The state of being underinformed can thus be easily overcome, such that the presence of search qualities should reduce the hesitation of consumers and thus increase the probability of video game success (Hennig-Thurau et al., 2001). Consequently, we expect search qualities to positively influence video game success and thus to constitute success factors in this regard. Regarding the identification of potential search qualities as video game success factors, findings from other entertainment sectors might appear transferable due to their objective nature.

Previous research on movie box office success highlights the important role of movie directors as a typical success factor. Within the motion pictures industry, the movie’s director has the task and ability to combine every creative aspect of a film and form it into a creative mix (Hennig-Thurau et al., 2007b). Taking this into consideration, the movie director’s tasks can be compared with the work of a developer in the gaming industry. Game development is a complex and creative process; thus, it requires rational structuring. For these tasks, a team of developers is responsible for designing and coding the game (Tschang, 2007). The existing research in the motion picture industry supports the idea that a developer has a positive influence on video game success. More specifically, movies created with more prominent directors are more popular and thus increase the chance of being more successful (Chen & Shugan, 2004; Moul, 2007; Zufryden, 2000). Transferring this into the video game industry leads to the proposition that games with more prominent developers are more popular and successful.

- H1: *The popularity of the developer has a positive impact on the success of the video game.*

In addition to the development team, the total size of the budget should also have an impact on game success. Previous research on motion pictures has demonstrated that large budgets positively influence a movie’s success, serving as a signal of good quality (Gemser et al., 2007; Prag & Casavant, 1994; Ravid, 1999; Zufryden, 2000). Consequently, based on the assumption that a video game player also perceives a high budget as a signal of good quality, the production costs of video games are expected to positively influence the financial success of a game.

- H2: *Production costs have a positive impact on the success of the video game.*

According to Hennig-Thurau et al. (2001), movies that include stars are more popular (see also Chang & Ki, 2005; De Vany & Walls, 1999; Skilton, 2009). Moreover, studies from Litman and Kohl (1989) and Bagella and Bechetti (1999) further underline the significant impact of star factor on movie success. Additionally, customers often count star factor within a motion picture as a signal of good quality (Arnold, 1992; Hennig-Thurau et al., 2012), leading to a positive impact on the movie’s revenue (Karniouchina, 2011). In recent years, “stars” in video games have also emerged, e.g., Lara Croft as the main hero of the Tomb Raider series (Griffiths et al., 2003), and so, star factor might also be a significant success factor in the video game industry.

- H3: *The inclusion of a star has a positive impact on the success of the video game.*

Past research on motion pictures provides strong evidence that there is a link between expert opinions and movie success (Eliashberg & Shugan, 1997; Wyatt & Badger, 1990). For instance, Hennig-Thurau and Wruck (2000) found that movie reviews have an impact on customers’ expectations and satisfaction after viewing the movie, contributing to the movie’s success. Similar to movie reviews, evaluations of video games by experts can be found online in databases such as metacritic.com or within gaming magazines (Hennig-Thurau et al., 2001; Ullmann et al., 2022). For players, they can be an aid in assessing game quality before buying the game (Cox, 2014) and thus are believed to have similar effects as movie critics (Hennig-Thurau et al., 2001).

- H4: *Reviews have a positive impact on the success of the video game.*

According to research conducted in the entertainment sector of motion pictures, the Oscars, as the most well-known movie awards, were shown to significantly influence movie success, with the category “best movie” shown to generate the most additional money (Deuchert et al., 2005; Dodds & Holbrook, 1988; Nelson et al., 2001). Within the video game industry, many awards are usually available for different categories, whereby the most well-known award is the “game of the year award” assigned by different institutions (British Academy Game Awards, Computer Bild Spiele, Deutscher Computerspielpreis, DICE Awards, Game Critics Awards, Game Developer Choice Awards, Spike VGX Awards). Since awards for video games have received increased attention in recent years, similar effects on video game success to those in the movie industry can be expected.

- H5: *Game awards have a positive impact on the success of the video game.*

In addition to these already mentioned success factors identified in other entertainment sectors, some video game-specific search qualities might also impact video game success. One of these search qualities is represented by the brand of the game (Cox, 2014). As the information published prior to the game’s release cannot be based on consumer experience, this kind of information is highly influenced by the producer brand itself (Marchand & Hennig-Thurau, 2013). Especially in the video game industry, the power of the brand influences the demand for games. Recent research results demonstrate that games with higher brand awareness, such as game series, are more successful than games from less popular brands (Marchand, 2016). Finally, existing research concerning the gaming industry confirms that only a few large players have the largest market share and are often both hardware and software producers (Marchand & Hennig-Thurau, 2013). Furthermore, blockbuster games are more likely to be published under a major brand (Cox, 2014) with higher brand awareness (Marchand, 2016) that also regularly includes more distribution resources and possibilities than minor brands (Elberse & Eliashberg, 2003; Hsu, 2006). Consequently, we propose to consider the popularity of the brand as an additional video game-specific success factor.

- H6: *The popularity of the brand has a positive impact on the success of the video game.*

Within the video gaming industry, there are commonly two types of developing strategies depending on the number of platforms the game is available for singlehoming and multihoming games (Marchand & Hennig-Thurau, 2013; Wiegand et al., 2022). Singlehoming is the choice of the developer to release the game on only one platform and thus foregoing money from buyers of other platforms (Landsman & Stremersch, 2011). It offers the platform producer a unique selling proposition, which, depending on the attraction of the game, can boost the importance of the platform (Venkatraman & Lee, 2004) and lead to increases in platform sales (Marchand & Hennig-Thurau, 2013). As a consequence, producers often pay developers an exclusive fee to compensate the developer for lost sales on other platforms (Landsman & Stremersch, 2011). On the other hand, multihoming refers to games being produced for more than one platform (Landsman & Stremersch, 2011; Marchand & Hennig-Thurau, 2013; Wiegand et al., 2022), reducing the differences between platforms for consumers and thus increasing sales for the game (Marchand & Hennig-Thurau, 2013; Venkatraman & Lee, 2004). Accordingly, a multihoming distribution strategy might act as a video game-specific success factor.

- H7: *The implementation of a multihoming distribution strategy has a positive impact on the success of the video game.*

Currently, video games often offer consumers the opportunity to play with other consumers via a multiplayer mode (Ullmann et al., 2022). Massive multiplayer online games such as World of Warcraft demonstrate that a strong multiplayer experience can foster the long-term loyalty of consumers and generate a constant revenue stream over a considerable number of years. World of Warcraft, which was introduced into the market in 2004, still has an active user base of approximately 8 million players even 18 years after market introduction. Accordingly, prior research refers to the multiplayer feature as an important value element for successful video games (Marchand & Hennig-Thurau, 2013). Consequently, we expect that the inclusion of a multiplayer mode represents an additional video game-specific success factor.

- H8: *The inclusion of a multiplayer mode has a positive impact on the success of the video game.*

**Fig. 1:**
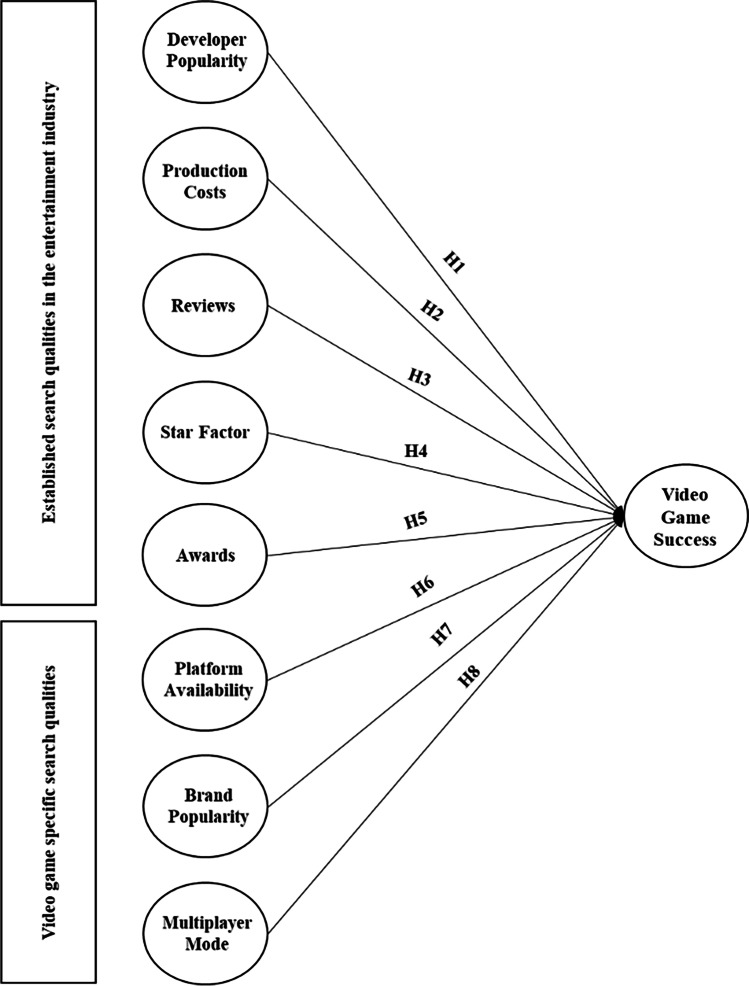
Conceptual model for search qualities

Figure 1 summarizes all hypotheses with regard to search qualities.

### Experience qualities and video game success

Economies of information theory suggests that information about experience qualities of video games (e.g., esthetics, ease of use, content) “have to be experienced by the consumer before he or she is able to evaluate them” (Hennig-Thurau et al., 2001, p. 2). Hence, the effort needed for acquiring information about experience qualities is higher compared to search qualities (Maute & Forrester, 1991). However, experience qualities are not subject to a seller bias or in need of verification through other sources, thus providing higher consumer benefits (Maute & Forrester, 1991). Hence, we also expect experience qualities to positively influence video game success and thus to constitute success factors in this regard. While for search qualities, it was possible to largely draw on previously identified success factors in neighboring entertainment industries, experience qualities have to be newly formulated for video games due to their unique usage experience that differs significantly from other entertainment products (Tavinor, 2008).

The first experience quality used to describe a video game usually refers to the presentation, i.e., aesthetic qualities of the game, meaning how the game looks and sounds to the player (King et al., 2010). Researchers assume that the utilization of graphical and acoustic effects in games makes the games appear more realistic and increases the level of immersion for the player (Boyle et al., 2012; King et al., 2010). Moreover, research has shown that players rate realistic qualitative sound and graphical elements as the most important aspect of video games that have a high impact on the user’s experience of the game (Nacke et al., 2010; Wood et al., 2004). As earlier research has clearly shown, the visual elements of a game influence the user experience (Nacke et al., 2010), and users rate high-quality graphics as an important influencing factor of game success (Wood et al., 2004). In line with Nacke et al. (2010), sound is defined as audio signals in digital games, including sound effects, character voices, and background music. Similar to the graphical aspects of a game, the sound shapes the user experience, guides the interaction within the game, and is often used as the main response interface for the user (Wood et al., 2004). According to previous research, good and realistic acoustic feedback increases the gameplay experience and is rated by users as one of the most important features of virtual games (Nacke et al., 2010; Wood et al., 2004).

- H9: A* game’s presentation has a positive impact on the success of the video game.*

Similar to presentation, ease of use has been identified as a key determinant for a successful gameplay experience and an increase in immersion (Hsu & Lu, 2004). Ease-of-use is defined as how quickly the player can get started with the game (Wood et al., 2004) and the effort it takes for the player to participate in the game in general (Wood et al., 2004). Previous research found that ease-of-use and interactivity are possible factors for the motivation to play games (Lin et al., 2012). More specifically, if a game is intuitive and the learning curve for the player is substantially decreasing over time, the player invests more time playing the game instead of learning how to do it, leading to an increase in immersion (Sweetser & Johnson, 2004). Ease-of-use in video games commonly refers to balance and game design. Balance relates to the “degree of cognitive and/or physical effort required in completing a task” (Orvis et al., 2008), whereas game design is defined as the controls or the interface between the game and the user (Tschang, 2005). On the one hand, balance takes into consideration that for players, challenging tasks and positive feedback are key factors in increasing both players’ satisfaction and the need to feel competent (Przybylski et al., 2010). Likewise, many players prefer games in which they can compete against other players (Liu et al., 2013). Previous research demonstrates that the motivation of a player is reduced if it is either too easy or too difficult, confirming that players experience greater immersion if their skills are equally balanced with the challenge at hand (Liu et al., 2013). On the other hand, the controller itself plays a critical role in the user experience of the game (Bianchi-Berthouze et al., 2007), as it acts as the direct link between the user’s actions and the game. Thus, controllers and game feedback are important elements of video games (Tschang, 2005).

- H10: *A game’s ease of use has a positive impact on the game’s success.*

Finally, the content of the video game, commonly encompassing campaigns, game background, or story as well as the games’ duration (Hastings, Guha and Stanley (2009), can be considered further experience quality driving success. Campaigns are defined as the sequence of events that appear during the game (Lin et al., 2012). They draw the player into the game and keep him or her linked to it (Lin et al., 2012). This is in line with the fact that games are often based on a story, film or book. For instance, the Harry Potter series started as a book was produced as a film and subsequently as a video game. Games based on such stories allow the player to expand their experience into a new area by allowing the player to adopt the role of a favorite character and engage with the game (Aoyama & Izushi, 2003). The first research into this area clearly confirms that introducing a story into a game leads to an increase in immersion and a greater identification with the game (Boyle et al., 2012). Moreover, researchers found that real-life settings were rated as important aspects of a game (Wood et al., 2004). Research within other entertainment sectors such as books and movies show that length is an important aspect to consider for the customer. Books are in general more successful the more pages they contain (Clement et al., 2006), whereas, for movies, a critical length exists. If a movie is too short, the customer believes that the movie is not worth the money they paid for it; however, if a movie is longer than a certain threshold, customers are often not willing to spend that much time watching it (Hennig-Thurau et al., 2001).

- H11: *A game’s content has a positive impact on the game’s success.*

**Fig. 2:**
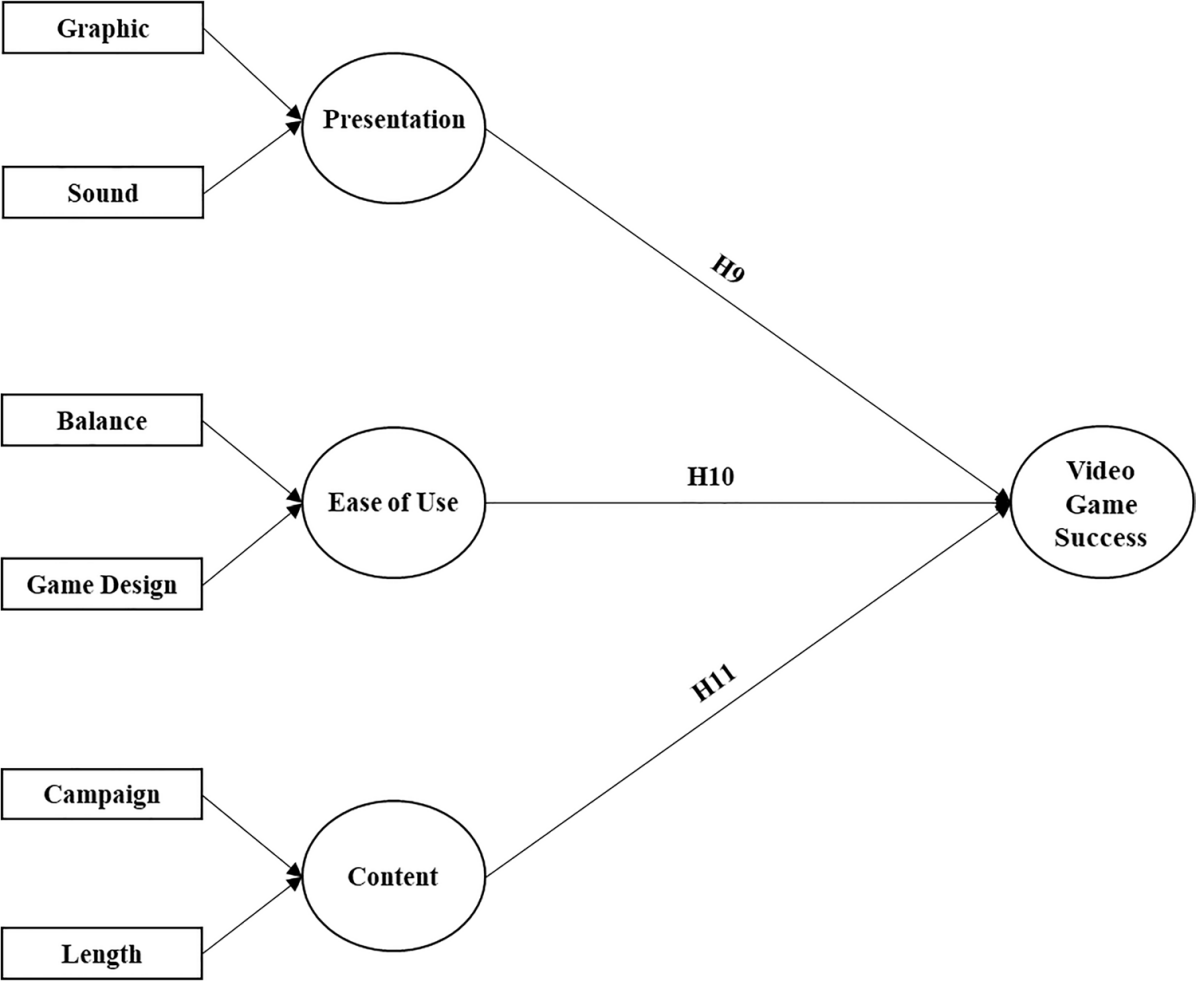
Conceptual model for experience qualities

Figure 2 summarizes all hypotheses with regard to experience qualities.

## Data and measurements

The sample consists of primary and secondary data on 351 computer games, including all games (successful and unsuccessful games in terms of items sold) that were published between 2010 and 2015 as well as reviewed in the largest (highest circulating) German game magazine “GameStar” (DWDL, 2016). Following prior research in the video game sector (Cox, 2014; Handrich et al., 2022; Situmeang et al., 2016), video game success was measured via sales figures. Following Cox (2014), measuring video game success through sales figures, in terms of all units sold in a specific period of time, offers a neutral and comparable base not affected by different exchange rates and different market prices in different European countries. More specifically, for each game, weekly sales figures for the first 3 years after the publication date of the game from the internet database vgchartz.com were collected and used as dependent variables. To further operationalize the dependent variable video game success, we followed procedures proposed by Handrich et al. (2022). Based on the assumption of Moore’s law that every 12 to 24 months a jump in technical performance is happening, Handrich et al. (2022) assume that on average, every 18 months new technical capabilities might initiate a new generation of games. In line with this theoretical reasoning and the operationalization of Handrich et al. (2022), we decided to utilize two success measurements as dependent variables reflecting two points in time. Short-term success is defined as the number of all sold units in Europe during the first 18 months after release, whereas long-term success is based upon all sold items during the second 18 months. With respect to search qualities, developer popularity was measured as the general awareness of different developer teams based on an expert evaluation, which is common practice in marketing and innovation research (Sweeney & Soutar, 2001; Zaichkowsky, 1985) as well as research on video games (Handrich et al., 2022). The two experts were chosen based on their experience with video games, i.e., at least 20 years of gaming experience, and a high level of involvement with video games (6.5 with 7 representing the maximum value) as well as previous memberships in e-sport clans or other communities in the video game sector. For each game, the leading developer team was collected from the online database igdb.com and evaluated by the two independent experts concerning the awareness of the developer based on a 5-point Likert scale anchored with totally unknown (1) to well known (5). The final value per game was generated by building the average of both expert opinions per developer team. To check for interrater reliability, we calculated the intraclass correlation coefficient (Shrout & Fleiss, 1979). The calculated intraclass correlation coefficient was 0.51, indicating moderate reliability (Koo & Li, 2016). Data on brand popularity were collected similarly to developer popularity. The general awareness of a game’s brand was measured based on expert evaluation. For each game, the main producer was identified (based on the online database igdb.net) and evaluated by two independent experts concerning the awareness of the publisher based on a 5-point Likert scale anchored with totally unknown (1) to well known (5). The final measurement was built using the average of both expert opinions per publisher. We again calculated the intraclass correlation coefficient (Shrout & Fleiss, 1979), indicating good reliability with a value of 0.72 (Koo & Li, 2016). Contrary to motion pictures where information concerning budgets is often publicly known, the data availability for games’ production costs is usually limited to blockbuster games (Economist, 2014). Nevertheless, game production costs for a number of different games could be derived from an article published on the German game website gameshardware.de (PC Games Hardware, 2016). Information about the star factor for each game was derived based on whether the name of the main character appeared in the game’s title. The number of awards won was collected from seven European or international game award websites (British Academy Award, Deutscher Computer Spiel Preis, DICE Award, Gamescom Award, Game Critics Awards, Spike Awards, Game Developer Choice Awards) for awards being handed out between 2010 and 2015. These seven awards were selected based on their awareness in the ranks of video game experts. Furthermore, information about the number of supported platforms per game was gathered from the internet database idgb.com. The star factor was operationalized as a binary variable where 1 (= known star factor) was given if the name of the main game character was part of the game’s title; otherwise, the value was set to 0. Regarding the search quality “reviews,” data on reviews published in game magazines as well as those published in-game databases were collected. For data on magazine reviews, the overall game value from the largest (highest circulating) German game magazine “GameStar” was collected, whereas the data on online reviews were selected from the online database “metacritic,” which, next to games, also evaluates other entertainment products. Both factors were measured on a 0 to 100 scale, with 100 being an excellent result, in accordance with both the metacritic-page (www.metacritic.com) and the GameStar review scale (Gamestar, 2015). The review measurement was calculated as the average of both factors. In addition, data on the number of supported platforms based on the information available on the database idgb.com were collected. Finally, for each game, information about the inclusion of a multiplayer mode was collected from the online database igdb.com. Afterward, a binary variable was coded where the value 1 was given if a multiplayer mode was part of the video game; otherwise, the value was set to 0.

With respect to experience qualities, review data from the highest circulating German Gaming magazine, namely, The GameStar (DWDL, 2016), were collected to establish the independent variables. More specifically, the review scheme of the magazine is based upon five different criteria—presentation (graphic and sound), balance, game design, atmosphere, and length—that were used to operationalize each experience quality. To make all items comparable, the items were standardized to a scale from 0 to 100.

## Analysis and results

For statistical analysis, multiple regression analyses using SPSS 27.00 were run for both research models by applying mean replacement as the missing value algorithm. For hypothesis testing, regression coefficients and their significances were assessed for each research model, further subdivided into a short-term and long-term success model.

### Research model on search qualities

As the research model on search qualities exclusively consists of constructs with a single indicator, we only checked for the discriminant validity of the constructs. As Table 1 of the Appendix shows, all construct correlations are well below common thresholds. To provide an even stronger test for discriminant validity, we also assessed the correlations’ heterotrait-monotrait ratio (HTMT) (Henseler et al., 2015). The HTMT values of all the construct pairs turned out to be below the more conservative threshold of 0.85 (the highest value of 0.673 between short-term and long-term success). Consequently, discriminant validity is given in the research model on search qualities.

**Table 1:** Results for search qualities

	Short-term success		Long-term success	
	Beta	*t* value	Beta	*t* value
Developer popularity	0.013	0.255	0.118	2.154
Production costs	0.001	0.018	− 0.072	1.421
Reviews	0.204	3.866	0.153	2.789
Star factor	− 0.056	1.132	0.008	0.154
Awards	0.180	3.512	0.111	2.095
Platform availability	− 0.098	1.919	− 0.099	1.884
Brand popularity	0.227	4.277	0.177	3.215
Multiplayer mode	0.115	2.316	0.101	1.980

As shown in Table 2, the dependent variables of short-term and long-term success were regressed on the predicting variables of developer popularity, production costs, reviews, star factor, awards, platform availability, brand popularity, and multiplayer mode. The independent variables significantly predict short-term success (*F*(8, 342) = 10.781, *p* < 0.01) and long-term success (*F*(8, 342) = 7.161, *p* < 0.01), indicating that all three factors have a significant impact on video game success. Furthermore, the data fit the model well, with an *R*^2^ of 0.201 for short-term success and 0.143 for long-term success. To check for multicollinearity, the variance inflation factor (VIF) was calculated for both dependent constructs. As the highest VIF value was 1.530, no multicollinearity exists (Henseler et al., 2009).

**Table 2:** Measurement model fit for experience qualities

Constructs	Item	Mean	SD	Weight	Significance (Bootstr. *n* = 5000)	VIF
Presentation	Graphic	69.84	22.154	0.625	3.516	1.878
	Sound	77.87	23.533	0.462	2.488	
Ease of Use	Balance	72.74	23.381	0.681	3.596	1.573
	Design	74.34	23.347	0.429	2.126	
Content	Campaign	69.75	26.125	0.196	1.195	1.257
	Length	74.80	26.206	0.896	9.220	

Regarding developer popularity as a search quality, our results show no significant effect on short-term success (*β* = 0.013, n.s. but a positive and significant effect on long-term success (*β* = 0.118, *p* < 0.05), partly confirming hypothesis 1. Contrary to our predictions in hypothesis 2, production costs did not significantly affect short-term success (*β* = 0.001, n.s.) nor long-term success (*β* =  − 0.072, n.s). In line with hypothesis 3, reviews positively affected both short-term (*β* = 0.204, *p* < 0.01) and long-term success (*β* = 0.153, *p* < 0.01). Star factor affected neither short-term success (*β* =  − 0.063, n.s.) nor long-term success (*β* = 0.008, n.s.), leading to a rejection of hypothesis 4. Confirming hypothesis 5, awards exhibited a positive effect on both short-term (*β* = 0.180, *p* < 0.01) and long-term success (*β* = 0.111, *p* < 0.05). Contrary to our predictions in hypothesis 6, platform availability negatively affected short-term success (*β* =  − 0.098, *p* < 0.10) as well as long-term success (*β* =  − 0.099, *p* < 0.10). In line with hypothesis 7, brand popularity positively affected both short-term (*β* = 0.227, *p* < 0.01) and long-term success (*β* = 0.177, *p* < 0.01). Finally, the inclusion of a multiplayer mode positively influenced short-term (*β* = 0.115, *p* < 0.05) and long-term success (*β* = 0.101, *p* < 0.05), confirming hypothesis 8.

### Research model on experience qualities

In the first step, we checked for discriminant validity of the constructs. As Table 3 of the Appendix shows, all construct correlations are well below common thresholds. We again assessed the correlations’ heterotrait-monotrait ratio (HTMT) (Henseler et al., 2015). The HTMT values of all the construct pairs were again below the more conservative threshold of 0.85 (the highest value of 0.673 between short-term and long-term success). Consequently, discriminant validity is also given in the research model on experience qualities. For each construct capturing experience qualities, two formative indicators were used for operationalization. Thus, the significances of all outer weights were evaluated using a bootstrapping algorithm (Chin, 1998; Tenenhaus et al., 2005). Table 4 shows that the significances of all formative indicators, except the item “campaign,” exceed the critical value of *t* > 1.98 (Chin & Newsted, 1999). For formative constructs, all items should be kept because otherwise the content of the construct might be altered (Götz et al., 2010), and we decided to keep the indicator “campaign” in the further analysis. In addition, we calculated the VIF for all items to assess multicollinearity. The highest VIF value was 1.878, showing that no multicollinearity exists (see Table 4) (Henseler et al., 2009). Based on the encouraging results of the operationalizations, a latent variable score was calculated for each construct and was subsequently used in the regression analysis.

**Table 3:** Results for experience qualities

	Short-term success		Long-term success	
	Beta	*t* value	Beta	*t* value
Presentation	0.072	1.048	0.179	2.674
Ease of use	− 0.022	0.305	− 0.007	0.103
Content	0.203	3.074	0.167	2.567

**Table 4:** Summary on managerial implications

Focus on	Success factor	Importance	Recommended actions
Experience qualities prior market introduction	Content	Positive effect on short-term and long-term success	Hire experienced and skilled developers Focus on storytelling Conduct extensive playtesting Incorporate player feedback
	Presentation	Positive effect on long-term success	Incorporate high-quality graphics and visual effects Create immersive and dynamic soundscapes Pay attention to detail
	Ease-of-use	No effect	Safe investments
Search qualities after market introduction	Reviews	Positive effect on short-term and long-term success	Highlight the unique benefits of the video game Offer incentives for reviews Engage with influencers
	Awards		Participate in game festivals Submit the game for awards Build a strong community
	Multiplayer mode		Include a multiplayer mode
	Brand popularity		Establish consistent branding Use cross promotion Host events Develop a strong online presence
	Developer popularity	Positive effect on long-term success	
	Star factor	No effect	Safe investments
	Production costs	No effect	Safe investments
	Number of platforms	Negative effect on short-term and long-term success	Employ a stepwise roll-out

As shown in Table 5, the dependent variables of short-term and long-term success were regressed on the predicting variables of presentation, ease of use, and content. The independent variables significantly predict short-term success (*F*(3, 347) = 6.663, *p* < 0.01) and long-term success (*F*(3, 347) = 11.071, *p* < 0.01), indicating that all three factors have a significant impact on video game success. Furthermore, the results confirm a sufficient fit of the estimations and data with an *R*^2^ for short-term success of 0.054 and for long-term success of 0.087. For both dependent constructs, the highest VIF value was 1.914. Hence, no multicollinearity should be present (Henseler et al., 2009). Consistent with hypothesis 9, the results show that presentation has a positive effect on the success of video games. However, the effect only appears significant for long-term (*β* = 0.179, *p* < 0.01) but insignificant for short-term success (*β* = 0.072, ns.). According to the results, hypothesis 10 is rejected, as the suggested positive impact of ease of use on both short-term (*β* =  − 0.022, n.s.) and long-term success (*β* =  − 0.007, n.s.) turned out insignificant. However, support was found for hypothesis 11, as content positively affected both short-term (*β* = 0.203, *p* < 0.01) and long-term success (*β* = 0.167, *p* < 0.01).

## Discussion

For an industry that is projected to grow by 7.17% (2023–2027), resulting in a market volume of US$482.30 bn in 2027 (Statista, 2023), it is surprising to only have suspicions but no empirical proof concerning the success factors of video games. Therefore, the research goal of this study was to identify potential success factors for video games, drawing on search and experience qualities as antecedent predictors of video game success. The results of the current empirical study have led to several noteworthy findings.

With regard to search qualities as antecedent predictors, the results confirmed that most of the potential success factors significantly affected either or both short-term and long-term success, with the most important search qualities in terms of highly significant and positive effect sizes being found to be reviews, awards, developer, and brand popularity as well as having a multiplayer mode. In general, this finding confirms that reviews, awards, and developer popularity, which were previously identified as success factors of other entertainment products such as motion pictures, also play an important role in video game contexts (cf. Eliashberg & Shugan, 1997; Gemser et al., 2007; Hennig-Thurau et al., 2001; Hennig-Thurau et al., 2006). Furthermore, with brand popularity and multiplayer mode, two additional success factors were identified for the context of video games.

However, in contrast to the motion picture industry, where the star factor has a positive impact on success (Bagella & Bechetti, 1999; Chang & Ki, 2005; De Vany & Walls, 1999; Litman & Kohl, 1989; Skilton, 2009), our analysis shows that star factor has no effect on video game success. One explanation for this result is that the player’s interaction with the game is at the core of the video game experience, while in motion pictures, the consumption of the product is closely linked to the performance of the actors (Tschang, 2005). Consequently, the focus of the player during game consumption is more on their performance and actions rather than on the possible star in the game. Likewise, our results also indicate that the production budget, which plays an important role in other entertainment industries such as motion pictures (Gemser et al., 2007; Prag & Casavant, 1994; Ravid, 1999; Zufryden, 2000), is not a success factor in the case of video games. This result might be because in the video game industry, only a few blockbuster games have high production costs with large returns on corresponding investments (Cox, 2014; Ullmann et al., 2022).

Another surprising finding is that the number of platforms a game is available for had a weak but negative effect on video game success. In line with the effect of the number of screens on movie success (De Vany & Walls, 1997; Hennig-Thurau et al., 2006; Neelamegham & Chintagunta, 1999; Sochay, 1994), a positive impact of a multihoming strategy was expected (Marchand & Hennig-Thurau, 2013; Venkatraman & Lee, 2004; Wiegand et al., 2022). A possible explanation for this effect might be that the focus of this analysis was computer games. Although computer games are also published on other platforms, such as consoles or mobile devices, the expected results of a multihoming strategy might be more prominent in the environment of game consoles, as the market is even more competitive because of the similar devices (Gao & Hallikainen, 2019; Shankar & Bayus, 2003).

With regard to experience qualities, all employed constructs were tailored to the peculiarities of the video game context and thus tested as success factors for this entertainment product for the first time. Within this respect, content and presentation emerged as the main success factors for video games. While content had a positive influence on both short-term and long-term success, the impact on short-term success was slightly higher than that on long-term success. A reason might be that at the time of market introduction, the storyline of the game is brand new and totally unknown. Consequently, a good storyline leads to higher success rates in the short-term perspective. Over time, increasingly more information concerning the story is available, e.g., on online forums, and so the impact on success decreases. Nevertheless, our findings confirm previous conceptual research suggesting that content is one of the main elements of video games (Wood et al., 2004). Contrary to content, presentation only had a significant impact on long-term success. An explanation might be that at the publishing date or shortly after, all new games have a state-of-the-art presentation. Especially in the same genre, the presentational aspects do not differ very much and therefore might not positively impact success. However, after some time, only games with superior presentational features, i.e., with realistic qualitative sound and graphical elements, can continue to exist in the market (Nacke et al., 2010; Wood et al., 2004).

Finally, and contrary to our expectation, ease-of-use does not influence either short-term or long-term game success. This finding might be because the perceived ease of use of a video game clearly depends on the genre and the player’s expectations toward the game. For instance, players of complex games such as MMORPGs expect more intricate game handling, while casual gamers prefer simpler game mechanics. Additionally, ease of use differs across player groups, as regular gamers have more experience than casual gamers, who play less frequently. Therefore, the impact of ease of use on success in video games depends on the type of game and the individual player.

## Theoretical implications

The findings of this research have produced several insights that might be relevant for theory and future research. This study is the first to provide empirical evidence on potential success factors in the video game industry. Our study thus answers calls made by Marchand and Hennig-Thurau (2013) as well as Pfau et al. (2022) to extend current research on success factors in the entertainment sector by investigations in the video game industry. The conceptualization of a typology of video game success factors based on the economics of information theory and empirical validation of search and experience qualities resulted in three major theoretical and two methodological contributions.

First, our study provides the first theoretical framework to classify and theoretically anchor the established and newly formulated success factors in entertainment industries such as video games. Based on the theory of the economics of information (Hirshleifer & Riley, 1992; Nelson, 1970; Stigler, 1961), we demonstrated the theoretical and empirical validity of categorizing video game success factors into search and experience qualities, as these factors have distinct conceptual differences and significantly differ in their relative importance in driving success. More specifically, according to the theory of economics of information, obtaining search and experience qualities for video games assists consumers in overcoming a state of being underinformed, leading to a positive appraisal in purchase decisions. While search qualities primarily provide consumer benefits due to their easy availability, experience qualities are not subject to a seller bias or in need of verification through other sources. Consequently, both types of video game qualities were proposed to be important success factors, which was empirically validated in our study accordingly. Our findings support Hennig-Thurau et al. (2001) theoretical propositions that economics of information theory provides unique insights into the mechanisms driving the success of entertainment products that are dominated by experience qualities. Nevertheless, our empirical validation provides the first empirical evidence for the transferability of the search and experience qualities classification scheme and corresponding theoretical rationales to the context of video games. Setting up this theoretical framework based on the economics of information theory is an important contribution for several reasons. The newly formulated framework represents a first step toward a theoretically grounded understanding of just what drives the success of entertainment products in general and of video games in particular. As mentioned before, different success factors have been studied in the entertainment industry more or less loosely side by side without any theoretical anchoring. However, a theoretical framework was necessary to establish a common ground for future empirical investigations into the success factors of the entertainment industry. This might enable comparisons of the relative importance of established constructs across research objects and areas and facilitate the integration of additional success factors to extend our current knowledge. The proposed framework intends to fill this gap and thus should facilitate further empirical investigations on success factors in the entertainment sector.

Second, while several studies have identified success factors for the motion picture industry, such as awards (Hennig-Thurau et al., 2001), star factor (Hennig-Thurau et al., 2012), and production costs (Gemser et al., 2007), the applicability and the transferability of these success factors beyond the context of movies and into other entertainment industries, such as video games has remained questionable. Our findings show that some of the established success factors in entertainment industries are indeed transferable to the context of video games, such as developer popularity, reviews, and awards. We thus add a new perspective for the applicability of established success factors in the entertainment industry, namely, the video game sector. In doing so, we not only replicate findings from the movie industry context but also confirm their applicability beyond the previously investigated contexts, which enhances their external validity. However, our findings also suggest that other established success factors, such as production costs and star factors, do not seem readily transferable to the context of video games. Possible explanations can be found in the peculiarities of the video game sector. Video games distinguish themselves from other forms of entertainment by seamlessly integrating audio-visual elements with sophisticated user engagement mechanisms (Tavinor, 2008). Our findings suggest that when studying success drivers in the entertainment industry, researchers should carefully consider the transferability of established success factors from adjacent research domains, such as the movie industry, to the domain of interest. They should also make adaptations to the underlying theoretical rationales if necessary.

Third, our findings reveal that the success factors for different entertainment products are unique and therefore require tailor-made approaches. To advance our understanding of potential success factors in the entertainment industry, cross-fertilization and literature convergence between similar research streams across different entertainment industries are valuable. However, due to the conceptual differences between entertainment products and the tight connection to different usage experiences of customers, it is necessary to derive and validate additional success drivers that are tailor-made for the peculiarities present in the respective entertainment sector. This especially accounts for success factors that refer to characteristics of entertainment products that can only be described after consumption. Future research on entertainment industry success factors should formulate new success factors based on the peculiarities of the investigated context if established success factors with experience qualities are not available.

Fourth, research concerning success factors in the motion picture industry as an example of the entertainment sector has a long history (Austin, 1981; Hadida, 2009; Hennig-Thurau et al., 2001, 2007b). However, research concerning success factors in the video game industry has been much more limited. More specifically, prior research has focused on issues such as how to succeed in gameplay (Trepte & Reinecke, 2011) or the influence of gaming on academic success (Anand, 2007), rather than the economic aspects of success factors in the video game industry. Wood et al. (2004) studied the factors that drive success for video games, but their approach was limited to the opinions of video game players and did not verify the results using established sales figures. Moreover, their online questionnaire was a snapshot of players’ opinions at one point in time, whereas success for entertainment products emerges due to a constant revenue stream over time. Hence, our research provides the first typology and empirical validation of the economic impact of several search and experience qualities as video game success factors over time.

Fifth, by taking a short-term and long-term perspective of video game success into account, differential impacts of success factors could be assessed over time. Specifically, some search qualities have stable effects on video game success over time, such as platform availability and multiplayer mode, while others exhibit significant variation in their impact on short-term versus long-term success. The effect of reviews, awards, and brand popularity turned out to be rather high from a short-term perspective but decreased over time. Thus, these search qualities may serve as gate openers to stimulate customers’ purchase behavior by signaling high quality, especially in the early stages of product release when consumers search for information before making a purchase decision. In contrast, developer popularity was found to be irrelevant from a short-term perspective but emerged as a significant success factor from a long-term perspective. This finding suggests that developer engagement, which tends to be higher for popular teams with greater resources, fosters interactions with player communities through co-creation of content and continuous updates (Kohler et al., 2011), resulting in a customer base that remains strong over time instead of diminishing. With respect to experience qualities, the effects of content turned out to be positive and rather stable for short-term and long-term success. However, presentation was only relevant as a success factor in the long-term perspective. This suggests that the experience qualities of entertainment products that heavily rely on acoustic and visual benefits during the usage experience may have a greater impact on customer loyalty in the long run than on initial purchases in the short run. Given the differential impacts of success factors on video games over time, future research should consider applying a more nuanced perspective with regard to success. Otherwise, effects that are only significant in the short term or long term might be missed.

## Managerial implications

Our results provide several managerial implications for video game producers and investors alike to better address the needs and requirements of game players. More specifically, as shown in Table 6, companies have the possibility to enhance video game success either (1) prior market introduction by adjusting experience qualities during video game development or (2) after market introduction by targeting search qualities within advertisements.

Prior market introduction companies should target experience qualities within new product development to enhance the suitability of their video games to current customer needs. According to our findings, content and presentation are the most important experience qualities of successful games. Concerning the content, it is important to include a compelling storyline or campaigns in the game (Handrich et al., 2022; Wood et al., 2004) and to ensure a certain gameplay duration (King et al., 2010; Wood et al., 2004). Therefore, companies can invest in creating engaging characters, plot twists, and narrative arcs that draw players in and keep them engaged throughout the game. A study from Schneider (2004) indicated that players preferred playing shooter games with a storyline, e.g., “Halflife” by Valve Software, over shooters without a story, e.g., “Doom 2” by id Software. To ensure the high quality of the storyline, companies should hire experienced and skilled developers who can create high-quality content. This can include game designers, writers, artists, and programmers who are passionate about creating engaging and immersive gaming experiences that also fit gamers’ expectations on gameplay duration. A good example of a successful game with a fitting game play duration is the game “Cyberpunk 2077,” which includes approximately 100 h of undisturbed playing time if a gamer strives to see all aspects of the game (Howlongtobeat, 2022). Furthermore, companies should conduct extensive playtesting and incorporate player feedback to make improvements to gameplay such that customer experience is enjoyable and engaging. Moreover, as graphics and sound are at the core of video games, investments in these two areas increase the probability of developing a successful game. In practice, producing games with state-of-the-art graphical elements might also increase the chance of producing a blockbuster game. For example, 3D technology or increased resolution technology such as 4 k are new technical opportunities that have enhanced the gameplay experience for games such as “Crysis 3” or “Assassin’s Creed 4.” To enhance the sound experience for users, some games now incorporate especially composed music or soundtracks from related media, such as motion pictures, as well as more realistic dialogs (Paterson et al., 2010). Managers could, for example, try to ask prominent musicians to have their music played in the background of a game, as was done in Rockstar’s “Grand Theft Auto V,” to increase realism in their games (NME, 2022). To enhance the user experience in games based on movies or TV shows, game developers can include specially composed music or soundtracks from the linked media and have the actual stars synchronize their characters in the game, as seen in the video game adaptation of the TV series “Game of Thrones,” where several actors lip-dub their characters (Golem, 2014). Similarly, prominent sports commentators can be brought in to act as commentators within sports games such as “FIFA 22” (Fourfourtwo, 2021). Finally, companies should fine-tune every aspect of the game’s presentation, from the user interface to the animations, to create a seamless and polished experience.

After market introduction, companies should use advertisements to enhance search qualities. One possibility in this respect is to focus on increasing the perceived quality of their products and thus of their brand. One way to increase perceived quality is to focus advertisement and promotional activities on the unique benefits the product offers compared to other brands (Balaji, 2011). For example, a strategic measure could be to highlight the development of strategic thinking for customers, in addition to the entertainment aspect of the video game. Another possibility is advertising with prominent testimonials or influencers (Spry et al., 2011). Here, especially for sport games, it might pay off to attract prominent athletes of the same sport to do the promotion. To boost the number of reviews and improve the overall rating of the game, companies may offer incentives to encourage players to leave reviews, such as free in-game content or exclusive access to new features. With respect to the strong effects of awards on video game success, it seems of the utmost importance that companies submit their games to relevant award shows and competitions. To enhance their chances of receiving an award, companies may participate in game festivals and conferences to showcase their games to a wider audience and network with industry professionals. Likewise, companies should build a strong community that might help in generating buzz and positive word-of-mouth for the game, which can increase the chances of receiving an award. Given the significant contribution of brand and developer popularity to video game success, companies with high brand recognition should establish a consistent branding for their video games, including logos, colors, and overall visual style. This creates a recognizable and distinctive brand that can help attract and retain players. To enhance brand and developer popularity, companies can cross-promote their games across different platforms and media. This can include advertising on social media, collaborating with influencers, and creating content such as trailers, gameplay videos, and merchandize. Likewise, companies may host events such as tournaments, in-game events, and meet-and-greets with developers. These events provide opportunities for players to engage with the brand and the developer, enhancing popularity while also generating buzz and positive word-of-mouth. Additionally, companies should establish a strong online presence to make it easy for potential consumers to acquire information about the brand and the developer. Last, companies should, if available, advertise the option to play the game in multiplayer mode, as this feature exhibited a strong effect on video game success.

In addition to these effective measures to boost game sales, the results of this study also demonstrate that certain search and experience qualities of video games may not be crucial or may even be detrimental to the success of the game. Search qualities such as star factors or production costs seem to not drive the success of video games as they do in other entertainment sectors such as the motion picture industry (Bagella & Bechetti, 1999; Hennig-Thurau et al., 2001; Litman & Kohl, 1989). Hence, companies should make safe investments in these areas and reconsider investing the corresponding amount in other search and experience qualities that turned out to be effective success factors. With respect to the multihoming distribution strategy, our results suggest that companies should carefully consider rolling out the video game on all available platforms simultaneously, as this might hamper sales numbers on certain platforms. Within this respect, it might be more beneficial to employ a stepwise roll-out on different platforms, beginning with the platform that potentially has the greatest potential to generate revenues.

## Limitations and directions for future research

This research is also subject to several limitations, which in turn provide avenues for further research. An avenue for future research could be to investigate the success factors in the console game industry, which might have distinct factors that are important to focus on. Currently, research in this area is limited, and including console games in the dataset could provide new insights into the unique success factors for this industry. Furthermore, another future research avenue in this respect would be to examine influencing factors for mobile games, which are a currently rising area of gaming (Gao & Hallikainen, 2019) and often include different aspects compared to computer games such as in-game selling and chance-based elements such as loot boxes (Adam et al., 2022).

Second, our data sample only consists of 351 games published between 2010 and 2015. Expanding the sample might also stabilize the results and generalize the findings. Furthermore, we incorporated video games from all around the world that were reviewed in the largest (highest circulating) German game magazine “GameStar” (DWDL, 2016) between 2010 and 2015. However, we relied on sales data from the internet database vgchartz.com that included the number of all sold items in Europe for 36 months after publication. While sales numbers of video games in Europe are commonly similar to those in other countries, i.e., successful video games in Europe are commonly also successful worldwide, we cannot exclude the possibility that for some video games, there might be an exception in this regard. Hence, it would be beneficial to examine the model not only with sales figures gathered for Europe but also for other regions worldwide, such as Asia, which has the largest growth rate concerning video games worldwide (Newzoo, 2015). As cultural differences exist between Asia and the Western world, it is possible that players in Asia place different importance on success factors compared to European players, as has been observed in the motion picture industry (Craig et al., 2005; Hennig-Thurau et al., 2007a). Therefore, it would be worthwhile for future research to explore whether success factors vary across different regions and cultures within the video game industry.

Third, we used only reviews from the most popular German game magazines for the corresponding analysis of experience qualities. While ratings in expert magazines are often quite similar, sometimes there are some differences as a result of subjective expert perception. Accordingly, future studies might validate our findings on the contribution of experience qualities to video game success using reviews from other game magazines and even from other countries.

Fourth, to formulate an initial theoretical framework for video game success factors with a high level of internal and external validity, we relied on secondary data from a period of time when external circumstances such as financial crises or pandemics did not drastically influence consumer purchasing or usage behavior. However, future research might draw on our theoretical framework and investigate whether generated findings during a rather stable time period might be affected by different external shocks that have taken place since that period, such as the COVID-19 pandemic. More specifically, it would be interesting to investigate whether the relative importance of different search and experience qualities may have changed during the COVID-19 pandemic. In doing so, our newly formulated framework would gain in external validity and be extended by potential boundary conditions.

Fifth, in our study, we strived to provide the most comprehensive picture of the potential success factors of video games. We thus investigated the impact of 8 search and 3 experience qualities on video game success. Although we included the most important established success factors from other entertainment sectors and derived a sufficient number of video game-specific success factors, there may be additional factors that were not considered in our analysis. Hence, future research may search for other success factors in the video game industry, empirically validate their effectiveness, and extend our theoretical framework by adding more search and experience qualities.

Sixth, success for entertainment products emerges due to a constant revenue stream over time, such that our main focus was to assess the economic impact of several search and experience qualities as video game success factors over time. Nevertheless, nonmonetary success dimensions are crucial areas to consider when investigating success factors, including effects on brand perception in terms of innovation and building a strong community of players who co-create and support the video game through updates. Future research might rely on our theoretical framework and investigate whether our identified search and experience qualities also account for nonmonetary success and whether their relative importance might vary compared to nonmonetary dependent variables.

Seventh, the main focus of our study was on investigating the success factors of video games. While identifying potential success factors is an important first step in understanding what makes a video game successful, it is also important to consider factors that may have a negative impact on success, such as errors or a lack of hotfixes. Future studies might therefore focus on barriers to video game success to complement the picture of what constitutes a successful video game.

Aside from the mentioned limitations, we suggest the following avenues for future research. First, future research could investigate whether the linkage to other media products, e.g., motion pictures or books, has an influence on the success of video games (Apperley, 2006; Hennig-Thurau et al., 2006). Second, another rewarding research avenue might be to consider game series (i.e., Grand Theft Auto, Need for Speed). In the case of video games, several game series exist and might result in new findings concerning the relevant aspects of these game series. Furthermore, the business model of mobile games is quite different from that of video games (MacInnes et al., 2002) since most mobile games employ a “free-to-play” (freemium) model to implement gameplay and game content (Gao & Hallikainen, 2019). Revenue is thus mainly generated by in-app purchases of content such as premium app updates (Liu et al., 2014) or virtual items or by viewing in-app advertising (Gao & Hallikainen, 2019; Hamari & Keronen, 2017). As a result, a primary challenge for mobile game developers is to retain their game players (Gao & Hallikainen, 2019). Consequently, it would be interesting to investigate in a long-term perspective whether the search and experience qualities identified as success factors in this study may differ with respect to their relative importance in mobile gaming contexts.

## Conclusion

In conclusion, this study provides a theoretical framework to classify and anchor success factors in entertainment industries, specifically video games, based on the economics of information theory. The study confirms the applicability of established search qualities from the movie industry context, such as developer popularity, reviews, and awards, as success factors in the video game sector. However, the study also suggests that other established success factors, such as production costs and star factors, do not seem readily transferable to the context of video games. This study further advances our understanding of potential success factors in the entertainment industry by demonstrating the need for tailor-made success factors for the respective entertainment context, especially with respect to experience qualities such as presentation and content. Overall, the resulting framework of search and experience qualities establishes a common ground that enables future empirical investigations into the success factors of the entertainment industry and provides new insights into the mechanisms that drive the success of entertainment products, which may guide future research in this field.

## Appendix

Please see Tables A.1 and A.2.

**Table A.1:** Descriptive statistics and correlation for the research model on search qualities

Construct	Mean	SD	1	2	3	4	5	6	7	8	9	10
1. Short-term success	134,537,313.39	2,124,59,748.62	1									
2. Long-term success	28,562,019.94	55,093,680.94	.673**	1								
3. Developer popularity	2.514	1.453	.139**	.204**	1							
4. Production costs	56,128,839.28	16,020,433.41	.016	− .053	.059	1						
5. Reviews	76.652	10.053	.304**	.234**	.206**	.014	1					
6. Star factor	0.091	0.028	− .083	− .016	− .013	.003	− .100	1				
7. Awards	0.455	1.319	.268**	.179**	.060	.007	.309**	− .049	1			
8. Platform availability	3.652	2.056	.004	.002	.211**	.040	.183**	.126*	.115*	1		
9. Brand popularity	3.720	1.316	.288**	.258**	.347**	.022	.179**	.097	.138**	.172**	1	
10. Multiplayer mode	0.621	0.485	.172**	.146**	.117*	.084	.112*	− .059	.012	.074	.151**	1

^**^Significant at *p* < 0.01, *significant at *p* < 0.05

**Table A.2:** Descriptive statistics and correlation for the research model on experience qualities

Construct	Mean	SD	1	2	3	4	5
1. Short-term success	134,537,313.39	212,459,748.62	1				
2. Long-term success	28,562,019.94	55,093,680.94	.673**	1			
3. Presentation	74.591	12.822	.161**	.260**	1		
4. Ease of use	74.850	15.323	.140**	.200**	.617**	1	
5. Content	75.898	17.196	.227**	.254**	.510**	.582**	1

^**^Significant at *p* < 0.01, *significant at *p* < 0.05
